# The Boltzmann Entropy and Randomness Tests

**DOI:** 10.3390/e28040429

**Published:** 2026-04-11

**Authors:** Peter Gács

**Affiliations:** Computer Science Department, Boston University, Boston, MA 02215, USA; gacs@bu.edu

**Keywords:** entropy, description complexity, Kolmogorov complexity, algorithmic entropy, randomness test

## Abstract

In the context of the dynamical systems of classical mechanics, we introduce two new notions called “algorithmic fine-grain and coarse-grain entropy”. The fine-grain algorithmic entropy is, on the one hand, a simple variant of the randomness tests of Martin–Löf (and others) and is, on the other hand, a connecting link between description (Kolmogorov) complexity, Gibbs entropy and Boltzmann entropy. The coarse-grain entropy is a slight correction to Boltzmann’s coarse-grain entropy. Its main advantage is its less partition-dependence, which is because algorithmic entropies for different coarse grainings are approximations of one and the same fine-grain entropy. It has the desirable properties of Boltzmann entropy in a wider range of systems, including those of interest in the “thermodynamics of computation”. It also helps explain the behavior of some unusual spin systems arising from cellular automata.

## 1. Introduction

This paper is an elaboration of the ideas proposed first in [1]. In its present form, the intended audience is mainly computer scientists interested in the relation of description complexity to thermodynamics. This determines the form of exposition somewhat: Section Appendix A gives a (very brief) introduction to some thermodynamical concepts and problems that physicists probably do not need. But we hope that the main technical content of this paper will ultimately be also considered a contribution to physics and dynamical system theory: mainly in providing some tools to extend thermodynamical reasoning to some new classes of systems.

The concept of computability is assumed to be known; for computability in continuous spaces, we refer to [2]. We also assume some familiarity with the notions of description (Kolmogorov) complexity. A good survey of the latter is [3].

The first version of this paper was written a long time ago. Among some developments on the topic that happened since, maybe the most important one is the paper [4], but see also the book [5].

### 1.1. Coarse-Graining

The physical model considered in this paper is that of classical mechanics: it is defined by a phase space Ω and a dynamics $U^{t}$ giving the point $U^{t}\omega$ on an orbit at time *t*, where the transformation $U^{t}$ preserves the volume measure *L* (like in Liouville’s Theorem; see Section Appendix A). In case of a container of “ideal” gas consisting of *n* simple molecules, the state space is the 6n-dimensional Euclidean space given by the positions and moments of each molecule.

A thermodynamical system is characterized by a relatively small number of parameters (functions of the state) called *macroscopic* parameters $u_{1},\ldots ,u_{m}$. The canonical example is a certain quantity of gas in a container with the macroscopic parameters of volume, temperature, energy and pressure. A microscopic state is the completely specified state. A macroscopic state is determined by the macroscopic parameters, and it determines only the (by far) most probable behavior and properties of the system and only when the system is in equilibrium.

We will assume that each macroscopical parameter $u_{i}$ takes only a finite number of values: a macroscopical parameter that is originally a real number will only be taken to a certain precision agreed in advance. This gives a partition of the state space into *cells*

$$\Omega =\underset{u}{⋃}\Gamma_{u}$$

corresponding to *macrostates* where $u=(u_{1},\ldots ,u_{m})$. Finer and finer partitions $P_{i}$ of the phase space can be introduced by adding more macroscopic parameters and more precision. The partition interpretation of a macrostate is called *coarse graining*.

Coarse graining solves the *paradox of irreversibility*: in a mechanical system, any evolution seems just as possible as the corresponding reverse evolution, and at the same time, the world seems to be full of irreversible phenomena (examples omitted). To reconcile the two pictures when we say that a certain transformation from state *a* to state *b* is reversible, this statement refers to *macrostates*; what is meant is that the reverse transformation exists for *most* microstates within the macrostate $\Gamma_{b}$, as measured by volume. Now, asymmetries are quite possible. It is easy to imagine macrostates a,b such that in a time unit, 99.99% of the elements of $\Gamma_{a}$ end up in $\Gamma_{b}$ but only 0.01% of the elements of $\Gamma_{b}$ end up in $\Gamma_{a}$. This is exactly what happens with the gas except that the percentages are much more extreme.

Another possible interpretation of a macrostate is as a certain distribution ν over microscopic states. It is possible (but not always necessary) to require ν to be a probability distribution, which is given by a density function p(ω) with ∫p(ω)L(dω)=1. Gibbs called such a distribution an *ensemble*. The ensemble $p_{\Gamma }\left(\omega \right)$ corresponding to a macrostate Γ is defined as 1/L(Γ) for ω∈Γ and 0 elsewhere.

### 1.2. Coarse-Grained Entropy

The Boltzmann entropy of a cell Γ is logL(Γ). This quantity depends on the choice of the partition: indeed, another digit of precision will decrease it by about log10. In the typical classical examples, this difference is negligible compared to the volumes in question. In nontypical systems, partition dependence can lead to paradoxes: we will show that the Maxwell demon paradox is one of these. We propose a new quantity

(1) $$S\left(\Gamma \right)=S_{L}\left(\Gamma \right)=K\left(\Gamma \right)+\log L\left(\Gamma \right)$$

called *coarse-grained algorithmic entropy* to replace Boltzmann entropy. Here, K(Γ) is the description (Kolmogorov) complexity of the cell Γ (to be defined later) (In earlier versions of this paper, we used the notation H(x) for K(x), but by now we are bowing to the general usage introduced in [3]). This quantity is closely related to the one introduced by Zurek, and we will return to their relation. The term K(Γ) is typically negligible compared to the Boltzmann entropy logL(Γ); however, the new entropy is less partition-dependent since it is an approximation to a certain partition-independent quantity $H_{L}\left(\omega \right)$, which we call (fine-grained) algorithmic entropy.

This paper explores the basic properties of fine-grained and coarse-grained algorithmic entropy. Description complexity itself will be shown to be a special case of algorithmic fine-grained entropy. The latter is a simple variant of the randomness tests of Martin–Löf (and others). Its integral over a Gibbs ensemble is close to the so-called Gibbs entropy. It possesses a simple conservation property that, together with coarse-grained algorithmic entropy, is helpful in explaining the Maxwell demon paradox as well as some other physical situations and models not handled well by Boltzmann entropy. Explanation of the entropy increase property for the new coarse-grained entropy does not rely on the huge volume differences in cells the way it does in the case of Boltzmann’s entropy.

We hope that the new quantity extends the possibilities of the kind of reasoning involving entropy to a wider range of systems (in particular, large computers in which it is not clear whether the whole memory content should be considered macroscopic or microscopic information). Here is an overview the content.

Section 2 defines description complexity K(x) (the self-delimiting version) of a finite objext *x* and shows its main properties.

Section 3 introduces randomness tests. The algorithmic (fine-grained) entropy $H_{\mu }\left(\omega \right)$ of a state ω of the system with respect to an underlying measure μ (typically, the volume measure over a finite-dimensional space or the counting measure over a discrete space) is defined as a common generalization of the description complexity K(x) and the randomness test of Martin–Löf (as modified by Levin). It is the negative logarithm of the maximal (to within an additive constant) lower semicomputable function f(x)≥0 with the property that

∫f(ω)μ(dω)≤1.

It is known that the description complexity K(x) is within constant distance from $H_{\#}\left(x\right)$ where # is the counting measure. The paper [6] has dealt with some statistical applications of this quantity.

Section 4 discusses an addition property for algorithmic entropy, which is the generalization of the information-theoretic addition property of complexity.

Section 5 expresses $H_{\mu }\left(\omega \right)$ in terms of complexity, with the help of a formula

K(Γ|μ)+logμ(Γ)

similar to (1).

In Section 6, we will prove a nondecrease property for algorithmic entropy. We give some examples that are better handled by our definition, which is partly because our entropy automatically satisfies the condition we call here the Noncompensation Condition. These examples do not belong to mainstream statistical mechanics but are borderline cases that may become more important with the further miniaturization of computers (“mesoscopic systems”?). It would also be interesting to see other chaotic systems in which the extension of the notion of entropy increase is useful.

Our definition resembles Zurek’s entropy defined in [7], but there are important differences. It is defined more generally (not just for systems consisting of a classical and a “demon” part). Its properties are proved with fewer assumptions. It connects randomness tests, fine-grain and coarse-grain entropy (these notions are not distinguished by Zurek) and complexity.

Heuristic arguments (and, of course, experience) show that in a nonequilibrium system, Boltzmann entropy can be expected to increase strongly. These arguments consist of an easy part, which we will call the Noncompensation Condition and a difficult part, the Weak Mixing Condition which, however plausible, can be rigorously proved only in some special cases. With our algorithmic Boltzmann entropy, the Noncompensation Condition comes free. The nontrivial, mixing part of the argument remains just as difficult and conditional as for Boltzmann entropy, but our framework allows at least to formulate the mixing property in a sharp way.

In Section 7, we will show an additivity property and will discuss its relation to the so-called Landauer thesis, which says that erasing a bit of information from, say, computer memory (even if it is performed indirectly) requires the dissipation of a certain minimal amount of heat. Maxwell’s demon paradox will also be alluded to in this context.

For the definitions of the various notions of computability in a continuous space (like the phase space of a system of particles), we refer to the lecture notes [2]. The concepts involved are quite technical, but it is possible to rely on an intuitive understanding of computability for the most part.

### 1.3. Notation

The sets of natural numbers, integers, rational numbers, and real numbers will be denoted, respectively, by N,Z,Q,R. The set of nonnegative real numbers will be denoted by $R_{+}$. We use ∧ and ∨ to denote min and max. In what follows, log is the logarithm to base 2. The quantities

⌊x⌋,⌈x⌉

are the largest integer ≤x and the smallest integer ≥x, respectively. For a set *E*, we denote the function taking the value 1 in *E* and 0 outside by $1_{E}\left(\omega \right)$, and we call it the *indicator function* of *E*. Borrowing from [8], for a two-variable function f(x,y) and a measure μ, we will sometimes use the notation

$$\mu f=\int f\left(x\right)\mu \left(dx\right),\;\mu^{y}f\left(x,y\right)=\int f\left(x,y\right)\mu \left(dy\right).$$

Let B={0,1}. We introduce a special object Λ called the “empty string”, and we define, as usual, the set of finite binary strings as

$$B^{*}=\left\{\Lambda \right\}\cup \overset{\infty }{\underset{n=1}{⋃}}B^{n}.$$

The set of infinite binary strings will be denoted by $B^{\omega }$. For a string $s\in B^{*}$, let

l(s)

denote its length.

## 2. Complexity

Given some computer *F*, let $K_{F}\left(y\right)$ be the length of the shortest program (measured in bits) that causes *F* to output string *y*. We will require the program to be self-delimiting: no endmarker is allowed. The machine-dependence of this concept is limited, since there is a machine *G* on which the function $K_{G}\left(y\right)$ is optimal within an additive constant: for every other machine *F*, there is a constant $c_{F}$ such that for all *x*, we have $K_{G}\left(x\right)\leq K_{F}\left(x\right)+c_{F}$. The notation

$$f\left(x\right)\overset{+}{<}g\left(x\right).$$

will mean that for some constant *c* and for all *x*, we have f(x)≤g(x)+c. The notation $\overset{*}{<}$ will mean the same with a multiplicative constant. The notation $\overset{+}{=}$ means that both $\overset{+}{<}$ and $\overset{+}{>}$ hold. With this notation, the invariance theorem’s formula can be written as $K_{G}\left(x\right)\overset{+}{<}K_{F}\left(x\right)$. The function $K\left(x\right)=K_{G}\left(x\right)$ is called the *complexity* of the natural number *x* conditional on the information *y*. (This is the modified version of the Kolmogorov–Solomonoff complexity invented by Levin and Chaitin. The book [3] uses the notation K(x) for the same quantity.) The conditional complexity K(x∣y) is defined by leaving *y* everywhere as a parameter. The elementary properties of the complexity function are discussed in several expositions, and we will not dwell on them. Let us just mention that $K\left(n\right)\overset{+}{<}\log n+2\log\log n$, and

(2) $$K\left(f\left(x\right)|y\right)\overset{+}{<}K\left(x|g\left(y\right)\right)\overset{+}{<}K\left(x\right).$$

for any computable functions f,g.

The function K(x) is not computable, but it has a certain weaker property. Let Q be the set of rational numbers. We call a function f(x) from natural numbers to real numbers *upper semicomputable* if there is a computable sequence $f_{n}\left(x\right)$ with rational values such that $f_{n}\left(x\right)↘f\left(x\right)$. It is easy to see that K(x∣y) is upper semicomputable. Thus, we can compute arbitrarily exact upper bounds on K(x), but we will not know how close we are to the limit. It is known that no nontrivial lower bounds can be computed for K(x). However, there are strong statistical lower bound results. There is a single property from which these can be derived:

$$\sum_{x}2^{-K(x|y)}\leq 1.$$

It is easy to prove this inequality by considering programs to our standard self-delimiting machine that are obtained by coin tossing. We will need the following important theorem of Levin.

**Theorem** **1** (Coding)**.** Let us consider the class of lower semicomputable functions f(x,y) with the property that $\sum_{x}f\left(x,y\right)\leq 1$. The function $2^{-K(x|y)}$ is an element of this class and is maximal in it to within a multiplicative constant. In other words, for each element f of this class, we have $2^{-K(x|y)}\overset{*}{>}f\left(x,y\right)$.

## 3. Randomness

We assume that the reader is familiar with the treatment of randomness tests in [2]. As there, following [9], we define a computable metric space as a tuple Ω=(Ω,d,D,α) where (Ω,d) is a metric space with a countable dense subset *D* and an enumeration α of *D*. It is assumed that the real function d(α(v),α(w)) is computable. As *x* runs through elements of *D* and *r* through positive rational numbers, we obtain the enumeration of a countable basis

β={Ball(x,r):x∈D,r∈Q}

(of balls or radius *r* and center *x*) of X, giving rise to a constructive topological space $\tilde{\Omega }$. Whenever we will have a transformation group $U^{t}$, we will assume that it as well as the invariant volume measure *L* are computable.

### Randomness Tests

A nonnegative lower semicomputable function $f_{\mu }\left(\omega ,y\right)$ over the space M(Ω)×Ω×Y is a (parametrized) *test of randomness* or, shortly, test with respect to a parameter *y*, if for all μ,y we have

$$\int f_{\mu }\left(\omega ,y\right)\mu \left(d\omega \right)\leq 1.$$

Here is some motivation for the case of probability measures. For a moment, forget the parameter *y*. If a certain casino claims that it draws elements from Ω according to the distribution μ, then it must accept the following deal:

- I prove that $f_{\mu }\left(\omega \right)$ is a test of randomness;
- I offer two dollars for a game and ask for ω;
- My payoff is $f_{\mu }\left(\omega \right)$.

If the casino owner indeed draws according to μ, then the test property implies that my expected payoff is at most a dollar, so she even makes more than a dollar of profit on average. My strategy is to try to find some nonrandomness in ω (without seeing ω first) that would make an acceptable test function $f_{\mu }\left(\omega \right)$ as large as possible. The following theorem is proved in [2], relying also on [10]:

**Theorem** **2.** *There is a universal test, that is, a test $t_{\mu }\left(\omega \right)$ with the property that for every other test $f_{\mu }\left(\omega \right)$, there is a constant $c_{f}>0$ with $c_{f}f_{\mu }\left(\omega \right)\leq t_{\mu }\left(\omega \right)$.*

The theorem can easily be generalized to include an extra parameter *y* in $f_{\mu }\left(\omega ,y\right)$. Then, it says that among all randomness tests, there is a certain one, denoted by $t_{\mu }\left(\omega |y\right)$ and called a *universal test*, that takes only values of the form $2^{n}$ for (possibly negative) integers *n* and is maximal to within a multiplicative constant: it has the property that for all other tests $f_{\mu }\left(\omega ,y\right)$, we have

$$f_{\mu }\left(\omega ,y\right)\overset{*}{<}t_{\mu }\left(\omega |y\right).$$

This test is a close relative of the universal tests of Martin–Löf and Levin and can be used as a criterion of randomness. (The property that t takes only values $2^{n}$ is only for convenience.) In the gambling interpretation, the universal test is, in some sense, optimal. Its existence is surprising: for example, if ω is supposed to be a sequence of coin tosses, then I could make my test function large for those ω’s in which the frequency of 1’s is at least 60% (since their probability is small). This way, I would profit from a certain kind of cheating the casino might attempt. Alternatively, I can make *f* larger on many other improbable sets of sequences. The universal test anticipates and combines all these strategies.

The *algorithmic (fine-grained) entropy* of ω with respect to μ is defined as

$$H_{\mu }\left(\omega |y\right)=-\log t_{\mu }\left(\omega |y\right).$$

We will delete μ from the subscript when it is obvious from the context. *H* can take arbitrarily large negative values, even −∞. In other words, an object can be infinitely nonrandom, although the measure of such objects has probability 0. For a finite measure μ, the function $H_{\mu }\left(\omega \right)$ is bounded from above. For infinite measures, it can also take arbitrarily large positive values, but it will never be *∞*.

Let $H_{\mu }\left(\omega \right)=H_{\mu }\left(\omega |0\right)$ where *Y* is chosen as the one-element set {0}. If both Ω and *Y* have measures μ,ν, then we define

$$H_{\mu ,\nu }\left(\omega ,y\right)=H_{\mu \times \nu }\left(\left(\omega ,y\right)\right)$$

where μ×ν is the product measure. To give some idea of how $H_{\mu }\left(\omega \right)$ depends on ω and μ, we give an upper bound. For $\omega \in \Omega_{n}\setminus \Omega_{n-1}$, let $m(\omega )=\mu (\Omega_{n})$. Then, it is easy to show that

$$H_{\mu }\left(\omega \right)\overset{+}{<}\log\left(m\left(\omega \right)+1\right)+2\log\left(\log\left(m\left(\omega \right)+1\right)+1\right).$$

Here are some additional easy properties of $H_{\mu }\left(\omega \right)$.

**Proposition** **1.**
(3) $$\begin{array}{cc} \mu \{\;\omega :H_{\mu }\left(\omega \right)<m\;\} & <2^{m}\;\left(-\infty <m<\infty \right), \end{array}$$

(4) $$\begin{array}{cc} H_{\nu }\left(y|\mu \right) & \overset{+}{<}-\log\int 2^{-H_{\mu ,\nu }\left(\omega ,y\right)}\mu \left(d\omega \right). \end{array}$$

*If f is a computable function, then*

$$H_{\mu }\left(\omega |y\right)\overset{+}{<}H_{\mu }\left(\omega |f\left(y\right)\right).$$

The first inequality states that $H_{\mu }\left(\omega \right)$ is large only with small probability. The second one is needed for an addition theorem stated later.

For the volume measure *L* in the phase space of a dynamical system, we define $H\left(\omega \right)=H_{L}\left(\omega \right)$ as the fine-grained algorithmic entropy of a state ω. Let

$$E_{m}=\left\{\;\omega :H_{L}\left(\omega \right)<m\;\right\}.$$

In (3), we have shown $L\left(E_{m}\right)<2^{m}$ for all *m*. For finite space volume L(Ω), this implies that

(5) $$L\left(E_{\log L(\Omega )-m}\right)/L\left(\Omega \right)<2^{-m},$$

that is, the proportial volume of the set of those points where algorithmic (fine-grained) entropy is not close to its maximum is very small. Let us note that the Coding Theorem 1 says

$$K\left(x|y\right)\overset{+}{=}H_{\#}\left(x|y\right)$$

where # is the counting measure. In this sense, the quantity $H_{\mu }\left(\omega \right)$ is a generalization of the complexity K(x). In what follows, whenever a measure is in the subscript, we will use the notation $H_{\mu }\left(x\right)$, but using it even in some other cases should not lead to any confusion. The following statement is similar to the universal uniform test theorem but for arbitrary measures:

**Proposition** **2.** *Let $\left(x,y,\nu \right)\mapsto f_{\nu }\left(x,y\right)$ be a nonnegative lower semicomputable function with $F_{\nu }\left(x\right)=\log\nu^{y}f_{\nu }\left(x,y\right)$. Then, for all x with $F_{\nu }\left(x\right)>-\infty$, we have*

$$H_{\nu }\left(y|x,\left\lfloor F_{\nu }\left(x\right)\right\rfloor \right)\overset{+}{<}-\log f_{\nu }\left(x,y\right)+F_{\nu }\left(x\right).$$

The proof is in [2].

## 4. Additivity

### Additivity of Information

Complexity has the following additivity property, which is due in various forms to Kolmogorov, Levin, Gacs, and Chaitin:

$$K\left(x,y\right)\overset{+}{=}K\left(y\right)+K\left(x|y,K\left(y\right)\right).$$

This property, in an appropriate form, generalizes for algorithmic entropy, as shown (in a different and slightly less general context) by Vovk and Vyugin:

**Theorem** **3** (Addition)**.**

$$H_{\mu ,\nu }\left(x,y\right)\overset{+}{=}H_{\nu }\left(y|\mu \right)+H_{\mu }\left(x|y,H_{\nu }\left(y|\mu \right),\nu \right).$$

Notice that this theorem differs from the original addition theorem for description complexity K(x) only by having μ,ν everywhere in the appropriate conditions and subscripts. (When μ is in the subscript, it does not have to be in the condition). The proof is in [2]. A simple corollary is

$$H_{\mu ,\nu }\left(x,y\right)\overset{+}{<}H_{\nu }\left(y\right)+H_{\mu }\left(x|y\right).$$

The function $H_{\mu }\left(\omega \right)$ behaves quite differently for different kinds of measures μ. For example, inequality (2) implies

$$K\left(y\right)\overset{+}{<}K\left(x,y\right).$$

In contrast, if μ is a probability measure, then

$$H_{\nu }\left(y\right)\overset{+}{>}H_{\mu ,\nu }\left(\omega ,y\right).$$

This comes from the fact that $2^{-H_{\nu }\left(y\right)}$ is a test for μ×ν. Due to these considerations, the following relation does not follow easily from the addition property (it would if $H_{\mu }\left(\omega \right)\overset{+}{<}H_{\mu \times \#}\left(\omega ,z\right)$ would hold): For *z* from a computably enumerable set *Y*, we have

(6) $$H_{\mu }\left(\omega \right)\overset{+}{<}H_{\mu }\left(\omega |z\right)+K\left(z\right).$$

This relation can be generalized slightly:

**Lemma** **1.** *For a computable function f(y,z) on a computably enumerable set Y, we have*

$$H_{\mu }\left(\omega |y\right)\overset{+}{<}H_{\mu }\left(\omega |f\left(y,z\right)\right)+K\left(z\right).$$

The proof is in [2]. Taking $f_{\mu }\left(x,y\right)=1$ in Proposition 2 gives the inequality

$$H_{\mu }\left(x|\left\lfloor \log\mu (X)\right\rfloor \right)\overset{+}{<}\log\mu \left(X\right),$$

with a physical meaning when μ is the phase space measure. Using (6), this implies

(7) $$H_{\mu }\left(x\right)\overset{+}{<}\log\mu \left(X\right)+K\left(\left\lfloor \log\mu (X)\right\rfloor \right).$$

The symmetric quantity I(x,y)=K(x)+K(y)−K(x,y) is called the *mutual information* between the objects x,y. It generalizes to

(8) $$\begin{array}{c} I_{\mu ,\nu }\left(x,y\right)=H_{\mu }\left(x|\nu \right)+H_{\nu }\left(y|\mu \right)-H_{\mu ,\nu }\left(x,y\right). \end{array}$$

(with respect to the measures μ,ν). A related quantity is

(9) $$\begin{array}{c} I_{\mu }\left(y:x\right)=H_{\mu }\left(x\right)-H_{\mu }\left(x|y\right), \end{array}$$

the *information* that *y* carries about *x* with respect to μ. When μ is obvious from the context, we omit it from the subscript. The complexity addition theorem is equivalent to the following relation between the two kinds of information:

$$I\left(x,y\right)\overset{+}{=}I\left(x,K\left(x\right):y\right)$$

which also generalizes to algorithmic entropy. The right-hand side can be interpreted as the information that the pair (x,K(x)) carries about *y*. Thus, I(x:y) is itself not symmetric but is “almost” symmetric since I(x,K(x):y) is equal to a symmetric quantity. More generally,

(10) $$\begin{array}{c} I_{\mu ,\nu }\left(x,y\right)\overset{+}{=}I_{\mu }\left(x,H_{\mu }\left(x|\nu \right):y|\nu \right). \end{array}$$

On a countable set *Y*, Lemma 1 implies

(11) $$I_{\mu }\left(y:x\right)\overset{+}{<}K\left(y\right).$$

This says that an object cannot carry more information about a string than its own complexity.

## 5. Randomness, Complexity, Coarse-Grained Entropy

### 5.1. Cells

As pointed out in [2], it is not convenient to define a measure μ constructively starting from μ(Γ) for open cells Γ. The reason is that no matter how we fix Γ, the function μ↦μ(Γ) is typically not computable. It is better to work with bounded computable functions, since for such a function *f*, the correspondence μ↦μf is computable. We therefore introduce a certain fixed, enumerated sequence of (Lipschitz) functions that will be used frequently. Let $F_{0}$ be the set of functions of the form $g_{u,r,1/n}$ where u∈D, r∈Q, n=1,2,…, and let

$$g_{u,r,\varepsilon }\left(x\right)={\left|1-|d\left(x,u\right)-r\right|}^{+}{/\varepsilon |}^{+}$$

be a continuous function that is 1 in the ball Ball(u,r), while it is 0 outside Ball(u,r+ε), and it takes intermediate values in between. Let

(12) $$F_{1}$$

be the smallest set of functions containing $F_{0}$ and the constant 1, and let it be closed under the Boolean operations: ∨, ∧ (that is max, min), and the complementation f↦1−f. Each element of the set $F_{1}$ is a continuous (actually, Lipschitz) function with values between 0 and 1. We could also call the set $F_{1}$ the set of *fuzzy cells*. Let E be the smallest set of functions containing $F_{1}$ and closed under the Boolean operations and also rational linear combinations. The following completeness property holds:

**Proposition** **3.** *All bounded continuous functions can be obtained as the limit of an increasing sequence of functions from the enumerated countable set E of bounded computable Lipschitz functions introduced above.*

The proof is routine. Under some special conditions, we will still obtain “sharp” cells. Let *f* be a bounded computable function over Ω, let $\alpha_{1}<\cdots <\alpha_{k}$ be rational numbers, and let μ be a computable measure with the property that $\mu f^{-1}\left(\alpha_{j}\right)=0$ for all *j*. In this case, we will say that $\alpha_{j}$ are *regular points* of *f* with respect to μ. Let $\alpha_{0}=-\infty$, $\alpha_{k+1}=\infty$, and for j=0,…,k, let $U_{j}=f^{-1}\left(\left(\alpha_{j},\alpha_{j+1}\right)\right)$. The sequence of disjoint r.e. open sets $\left(U_{0},\ldots ,U_{k}\right)$ will be called the *partition generated by* $f,\alpha_{1},\ldots ,\alpha_{k}$. If we have several partitions $\left(U_{i0},\ldots ,U_{i,k}\right)$, generated by different functions $f_{i}$ (i=1,…,m) and different regular cutoff sequences $\left(\alpha_{ij}:j=1,\ldots ,k_{i}\right)$, then we can form a new partition generated by all possible intersections

$$V_{j_{1},\ldots ,j_{n}}=U_{1,j_{1}}\cap \cdots \cap U_{m,j_{m}}.$$

A partition of this kind will be called a *regular partition*. The sets $V_{j_{1},\ldots ,j_{n}}$ will be called the *cells* of this partition. It is easy to see that the values $\mu V_{j_{1},\ldots ,j_{n}}$ are computable from the names of the functions $f_{i}$ and the cutoff points $\alpha_{ij}$.

From now on, we will assume that a computable sequence of functions $b_{1}\left(\omega \right),b_{2}\left(\omega \right),\ldots$ over Ω is fixed with the property that for every $\omega_{1},\omega_{2}\in \Omega$ there is a *j* with $b_{j}\left(\omega_{1}\right)<0$, $b_{j}\left(\omega_{2}\right)>0$. Let us give the correspondence between the set $B^{\omega }$ of infinite binary sequences and elements of the set

$$\Omega^{0}=\left\{\;\omega \in \Omega :b_{j}\left(\omega \right)\neq 0,\;j=1,2,\ldots \;\right\}.$$

For a binary string $s_{1}\cdots s_{n}=s\in B^{*}$, let

$$\Gamma_{s}$$

be the set of elements of Ω with the property that for j=1,…,n, if $s_{j}=0$, then $b_{j}\left(\omega \right)<0$; otherwise, $b_{j}\left(\omega \right)>0$. This correspondence has the following properties.

- $\Gamma_{\Lambda }=\Omega$.
- For each s∈B, the sets $\Gamma_{s0}$ and $\Gamma_{s1}$ are disjoint and their union is contained in $\Gamma_{s}$.
- For $\omega \in \Omega^{0}$, the set $\left\{\;\Gamma_{s}:\omega \in \Gamma_{s}\;\right\}$ forms a basis of its neighborhoods.

If *s* has length *n*, then $\Gamma_{s}$ will be called a *canonical n-cell*, or simply canonical cell, or *n*-cell. From now on, whenever Γ denotes a subset of Ω, it means a canonical cell. We will also use the notation

$$l\left(\Gamma_{s}\right)=l\left(s\right).$$

The three properties above say that if we restrict ourselves to the set $\Omega^{0}$, then the canonical cells behave just like binary subintervals: they divide $\Omega^{0}$ in half, then each half again in half, etc. Moreover, around each point, these canonical cells become “arbitrarily small”. It is easy to see that if $\Gamma_{s_{1}},\Gamma_{s_{2}}$ are two canonical cells, then they either are disjoint or one of them contains the other. If $\Gamma_{s_{1}}\subset \Gamma_{s_{2}}$, then $s_{2}$ is a prefix of $s_{1}$. If, for a moment, we write $\Gamma_{s}^{0}=\Gamma_{s}\cap \Omega^{0}$, then we have the disjoint union $\Gamma_{s}^{0}=\Gamma_{s0}^{0}\cup \Gamma_{s1}^{0}$. For $\omega \in \Gamma_{s}$, we will write

$$\omega^{n}=\omega_{1}\cdots \omega_{n}.$$

Thus, for elements of $\Omega^{0}$, we can talk about the *n*-th bit $\omega_{n}$ of the description of ω: it is uniquely determined. The $2^{n}$ cells (some of them possibly empty) of the form $\Gamma_{s}$ for l(s)=n form a partition

$$P_{n}$$

of $\Omega^{0}$. Let M(Ω) be the set of nonnegative measures μ over Ω with the property that μ(Ball(x,r))<∞ for all x,r. Let $M^{0}\left(\Omega \right)$ be the set of measures μ∈M(Ω) with the property that for each *n*, $\mu b_{n}^{-1}\left(0\right)=0$. For these measures μ, the functions $b_{j}$(j≤n) and the cutoff point 0 form a regular partition of Ω as defined above.

**Example** **1.**

*1.*  *If* Ω *is the set of infinite binary sequences with its usual topology, the functions $b_{n}\left(\omega \right)=\omega_{n}-1/2$ generate the usual cells, and $\Omega^{0}=\Omega$.*
*2.*  *If* Ω *is the interval [0,1], let $b_{n}\left(\omega \right)=-\sin\left(2^{n}\pi \omega \right)$. Then, cells are open intervals of the form $\left(k\cdot 2^{-n},\;\left(k+1\right)\cdot 2^{n}\right)$, and the correspondence between infinite binary strings and elements of $\Omega^{0}$ is just the usual representation of ω as the binary decimal string $0.\omega_{1}\omega_{2}\ldots$.*

When we fix canonical cells, we will generally assume that the partition chosen is also “natural”. The bits $\omega_{1},\omega_{2},\ldots$ could contain information about the point ω in decreasing order of importance from a macroscopic point of view. For example, for a container of gas, the first few bits may describe, to a reasonable degree of precision, the amount of gas in the left half of the container, the next few bits may describe the amounts in each quarter, the next few bits may describe the temperature in each half, the next few bits may describe again the amount of gas in each half, but now to more precision, etc. For elements of $\Omega^{0}$, we can talk about the *n*-th bit $\omega_{n}$ of the description of ω: it is uniquely determined.

### 5.2. Characterizing Tests via Complexity

This section is restricted to finite measures. Let $M_{R}\left(\Omega \right)$ be the set of measures μ with μ(Ω)=R. Theorem 1 (the Coding Theorem) implies that if *x* runs over a discrete space, then $H_{\#}\left(x\right)\overset{+}{=}K\left(x\right)$. More generally, it is shown in [2] that

(13) $$\begin{array}{c} H_{\mu }\left(x\right)\overset{+}{=}K\left(x|\mu \right)+\log\mu \left(x\right). \end{array}$$

Here, the function K(x|μ) must be defined appropriately as an upper semicomputable function of the pair (x,μ). There is a similar characterization of tests over arbitrary spaces. Let us denote

(14) $$\begin{array}{c} S_{\mu }\left(\Gamma \right)=K\left(\Gamma |\mu \right)+\log\mu \left(\Gamma \right) \end{array}$$

for canonical cells Γ. The following theorem is proved in [2]:

**Theorem** **4.** *Suppose that the space X is compact. Then, for all computable measures $\mu \in M_{R}^{0}\left(X\right)$, we have*

(15) $$H_{\mu }\left(\omega \right)\overset{+}{=}\underset{n}{\inf}S_{\mu }\left(\omega^{n}\right)).$$

*The constant in $\overset{+}{=}$ depends on the computable measure μ.*

The $\overset{+}{<}$ part of the statement is valid in a more general space and without assuming computability or compactness. Assume that a separating sequence $b_{1},b_{2},\ldots$ is given as in Section 5.1 along with the set $\Omega^{0}$. For each $\omega \in \Omega^{0}$, the binary sequence $\omega_{1},\omega_{2},\ldots$ has been defined. Let $M_{R}^{0}\left(\Omega \right)$ be again the set of those measures μ with $\mu (\Omega \setminus \Omega^{0}=0$. The following statement has also been proved in [2]:

**Proposition** **4.** *For all measures $\mu \in M_{R}^{0}\left(\Omega \right)$, we have*

(16) $$H_{\mu }\left(\omega \right)\overset{+}{<}\underset{n}{\inf}\left(\log\mu \left(\omega^{n}\right)+K\left(\omega^{n}|\mu \right)\right).$$

**Remark** **1.**

*1.*  *In (15), since we assumed μ is computable, we can actually delete it from the condition in the complexity. We left it there only since we would really like to prove that this equation holds uniformly over $M(\Omega^{0})$ and not only for computable measures.*
*2.*  *An interesting application where a related formula is used is the “minimum description length” principle (MDL) in the theory of statistics. There, instead of the description complexity K(x), often the codeword length C(x) of some other coding (universal over some class of measures) is considered, and the quantity C(Γ)+logμ(Γ) is called the redundancy. In these statistical applications, the presence or absence of μ in the condition makes a difference.*

Theorem 4 says that the fine-grained algorithmic entropy $H_{L}\left(\omega \right)$ with respect to the invariant volume measure *L* can be essentially expressed as $S\left(\omega^{n}\right)=K\left(\omega^{n}\right)+\log L\left(\Gamma_{\omega^{n}}\right)$ for a certain *n*: so, it is the sum of the Boltzmann entropy of the part $\Gamma_{\omega^{n}}$ plus the description complexity of the macroscopic description $\omega^{n}$. For the systems and partitions of interest in physics, the additive term $K\left(\omega^{n}\right)\overset{+}{<}2n$ is typically negligible compared to the other one, since the total number of macroscopic cells is typically small compared to the volume of the large cells. In order to find the “right” *n*, we might keep increasing it, including (the program for) more and more bits of ω into the macroscopic description as long as the complexity increase buys a greater decrease in the Boltzmann entropy $\log L(\Gamma_{\omega^{n}})$ (our a priori uncertainty about ω).

The following theorem says that $H_{\mu }\left(\omega \right)$ cannot be much higher than $S_{\mu }\left(\Gamma \right)$ for most elements ω of a cell Γ.

**Theorem** **5** (Stability)**.**

$$\mu \left\{\;\omega \in \Gamma :H_{\mu }\left(\omega \right)<S_{\mu }\left(\Gamma \right)-K\left(l\left(\Gamma \right)\right)-m\;\right\}\overset{*}{<}2^{-m}\mu \left(\Gamma \right).$$

We can also interpret this theorem as saying that the randomness of most elements of a cell is near their maximum within the cell. Note that the difference K(l(Γ)) is less than 2logn for $\Gamma_{\omega^{n}}$.

**Proof.**  Let $f\left(\Gamma \right)=2^{-K(l(\Gamma ))}\int_{\Gamma }2^{-H_{\mu }\left(\omega \right)}\mu \left(d\omega \right)$. This function is semicomputable and $\sum_{\Gamma }f\left(\Gamma \right)\leq 1$. Therefore, $f\left(\Gamma \right)\overset{*}{<}2^{-K(\Gamma )}$. Rearranged, this gives:

$$\mu {\left(\Gamma \right)}^{-1}\int_{\Gamma }2^{-H_{\mu }\left(\omega \right)}\mu \left(d\omega \right)\overset{*}{<}2^{-K(\Gamma )-\log\mu (\Gamma )+K(l(\Gamma ))}\;\;\;=2^{-S_{\mu }\left(\Gamma \right)+K\left(l\left(\Gamma \right)\right)}.$$

From here, we conclude with Markov’s inequality. □

Let $i_{n}\left(\omega \right)$ be the *i* where the minimum is achieved. As a function of *n*, for certain ω, this may make a sudden jump from a much smaller value to *n*, suggesting instability in the coarse-grained quantity. However, there is another interpretation. In general, what is given is a canonical cell Γ. Theorem 5 (the Stability Theorem) implies

$$L\left\{\;\omega \in \Gamma :H_{L}\left(\omega \right)<S_{L}\left(\Gamma \right)-m-K\left(l\left(\Gamma \right)\right)\;\right\}\overset{*}{<}2^{-m}L\left(\Gamma \right).$$

In other words, for most elements ω of the canonical cell Γ, the value $H_{L}\left(\omega \right)$ is not much less than $S_{L}\left(\Gamma \right)$. In a sense, a string $\omega^{n}$ describing a canonical cell never has too few bits since it is always a nearly optimal description for most states in it. But it may have too many bits in the sense that deleting some of the last ones results in a significant decrease of the entropy of the canonical cell: the deletion decreases the complexity with only a smaller increase in logL.

## 6. Entropy Increase Properties

### 6.1. Fine-Grain Nondecrease

As stated in the Introduction, we are considering an isolated physical system with state space Ω whose development is described by a transformation group $U^{t}$. We also assume that $U^{t}\omega$ is computable as a function of the pair ω and *t*. We assume the existence of a computable invariant measure *L* (the “Liouville measure”): it obeys $L\left(U^{t}A\right)=L\left(A\right)$ for all *t* and all measurable sets *A*. Under suitable conditions, the existence, computability and even uniqueness of *L* can be proven. For each *t*, since $U^{t}$ is measure-preserving, the function $2^{-H_{L}\left(U^{t}\omega \right)}$ is a parametrized randomness test, hence

$$H_{L}\left(U^{t}\omega \right)\overset{+}{>}H_{L}\left(\omega |t\right)=H_{L}\left(\omega \right)-I_{L}\left(t:\omega \right),$$

where $I_{L}\left(t:\omega \right)$ was defined in (9). This is, in essence, our entropy nondecrease formula, since we will see that the term $I_{L}\left(t:\omega \right)$ is generally very small. It can also be regarded as a special case of a more general randomness-conservation property formulated by L.A. Levin in several ways, see for example [11]. (Other forms can be found in [2,12]). Alas, it is also an entropy nonincrease formula when applied to $U^{-t}$. Therefore, we obtain

(17) $$-I_{L}\left(t:\omega \right)\overset{+}{<}H_{L}\left(U^{t}\omega \right)-H_{L}\left(\omega \right)\overset{+}{<}I_{L}\left(t:U^{t}\omega \right).$$

According to this, the only amount of decrease we will ever see in $H_{L}\left(U^{t}\omega \right)$ is due to the information that the value of the time *t* may have on ω, which is very small for all simple moments of time. But the amount of increase is also only due to the information that *t* may have on $U^{t}\omega$. Let us explore the nondecrease property.

**Theorem** **6** (Entropy Nondecrease)**.** *Let λ be the length (Lebesgue) measure, and let T be a rational value of time. We have*

$$\lambda \left\{\;t\in \left[0,T\right]:H_{L}\left(U^{t}\omega \right)<H_{L}\left(\omega \right)-K\left(T\right)-m\;\right\}\overset{*}{<}2^{-m}T.$$

This theorem follows, by an application of Markov’s Inequality, from the following lemma.

**Lemma** **2.**
$$T^{-1}\int_{0}^{T}2^{I_{L}\left(t:\omega \right)}dt\overset{*}{<}2^{K(T)}.$$

**Proof.**  The function $f\left(\omega \right)=T^{-1}\int_{0}^{T}2^{-H_{L}\left(\omega |t\right)}dt$ is a randomness test and therefore

$$-\log f\left(\omega \right)\overset{+}{>}H_{L}\left(\omega |T\right)\overset{+}{>}H_{L}\left(\omega \right)-K\left(T\right)$$

(using Lemma 1). Hence,

$$T^{-1}\int_{0}^{T}2^{H_{L}\left(\omega \right)-H_{L}\left(\omega |t\right)}dt\overset{*}{<}2^{K(T)}$$

from where we conclude which by the definition of $I_{L}$. □

### 6.2. Gibbs Ensembles

Another accepted model of a macrostate is a certain distribution ν over microscopic states given by a density function (ensemble) p(ω) with respect to the volume measure *L* over the space Ω. Let us require ∫p(ω)L(dω)=1. We can ask what is the probability density to find the system in state ω at time $t+t_{0}$? Let us call this new ensemble $p^{t}$. The classical definition of Gibbs entropy of a probability distribution with density function p(ω) over *L* is

G(p)=−∫p(ω)logp(ω)L(dω).

In the special case when *p* is the ensemble describing macrostate Γ (that is a positive constant on Γ and 0 everywhere else), we have $G\left(p_{\Gamma }\right)=\log L\left(\Gamma \right)$; that, is the Gibbs entropy is the same as the Boltzmann entropy. Liouville’s Theorem implies $G\left(p^{t}\right)=G\left(p\right)$, that is the Gibbs entropy of an ensemble does not change at all in an isolated system during evolution.

This shows that in case of the evolution of isolated nonequilibrium systems, the evolution of a Gibbs ensemble does not express adequately what we consider thermodynamic behavior. The problem is that even if at the starting time $t_{0}$ the Gibbs ensemble was something simple, it can develop in time *t* into a very complicated density function that does not correspond to any reasonable macroscopic description. Ensembles that are invariant in time retain their usefulness, however, for equilibrium systems.

Let us relate Gibbs entropy to the average of algorithmic entropy. Let q(x)>0 be another density function. Let a=∫p(ω)L(dω) (which we assumed to be 1), and b=∫q(ω)L(dω). The well-known inequality (see for example [2]), which requires no computability assumptions, says

$$\begin{array}{c} \int p\left(\omega \right)\log\frac{p(\omega )}{q(\omega )}L\left(d\omega \right)\geq a\log\frac{a}{b}. \end{array}$$

This can be written as

$$\begin{array}{c} G\left(p\right)=-\int p\left(\omega \right)\log p\left(\omega \right)L\left(d\omega \right)\leq -\int p\left(\omega \right)\log q\left(\omega \right)L\left(d\omega \right)-a\log\frac{a}{b}. \end{array}$$

Let us choose now $q\left(\omega \right)=2^{-H_{L}\left(\omega \right)}$, then a=1, b<1, giving

(18) $$\begin{array}{c} G_{L}\left(p\right)<-\int H_{L}\left(\omega \right)p\left(\omega \right)L\left(d\omega \right); \end{array}$$

that is, the Gibbs entropy is always less than the average algorithmic entropy. On the other hand, now using the computability of p(ω), we can view p(ω) as a randomness with respect to the probability measure dL/L(Ω), and therefore it is majorized by $t_{L}\left(\omega \right)$ with a factor depending only on the complexity K(p) of *p*. Going to logarithms, we can write

$$\begin{array}{c} -\log t_{L}\left(\omega \right)=H_{L}\left(\omega \right)\overset{+}{<}-\log p\left(\omega \right)+K\left(p\right). \end{array}$$

It follows that

(19) $$\begin{array}{c} G_{L}\left(p\right)<-\int H_{L}\left(\omega \right)p\left(\omega \right)L\left(d\omega \right)\overset{+}{<}G_{L}\left(p\right)+K\left(p\right). \end{array}$$

In other words, the Gibbs entropy is both lower-bounded and upper-bounded by the average algorithmic entropy. Let us express the randomness test for the measure ν whose density function is *p* by the randomness test for *L*.

**Theorem** **7.**
$$H_{\nu }\left(\omega \right)\overset{+}{=}H_{L}\left(\omega \right)+\log p\left(\omega \right)$$

*where the constant in $\overset{+}{=}$ depends on the definition of the function p(ω).*

**Proof.**  For an arbitrary lower semicomputable function f(ω,ν), we have

∫f(ω,ν)ν(dω)=∫f(ω,ν)p(ω)L(dω).

Therefore, *f* is a test for ν if and only if fp is a test for *L*. The maximal *f* will therefore be $\overset{*}{=}\;2^{-H_{L}\left(\omega \right)+\log p\left(\omega \right)}$. □

Notice that the theorem does not allow to compute $H_{L}\left(\omega \right)$ from $H_{\nu }\left(\omega \right)$ in the places where p(ω)=0.

### 6.3. The Increase of Coarse-Grained Entropy

Let us now consider the much more speculative problem of approach to equilibrium: the argument that the algorithmic Boltzmann entropy $H^{n}\left(U^{t}\omega \right)$ must indeed increase fast if it is far from its upper bound logL(Ω). The problem of proving entropy increase mathematically has a long history without any satisfactory solution for the model of deterministic development that we are considering here. If we build randomness into the system’s evolution, assuming a Markov process, then entropy increase is easy to prove. For an application of the Markovian assumption to our framework here, see [4]. The difficult problem in the deterministic setting (well, deterministic evolution from a—somewhat—random initial condition) is to see, for example, how the movement of a particle in the future is becoming independent from that of other particles with which it interacted in the past. See, for example, one of the latest efforts to derive the Boltzmann equation from kinetic theory in [13].

There is a classical argument to show that in a nonequilibrium system, Boltzmann entropy can be expected to increase fast until it almost reaches its upper bound logL(Ω). The argument relies on two properties that I will try to formulate now. Let us look at systems in which

M=logL(Ω)

is a parameter: the maximal value of the entropy. We will not try to require convergence of the entropy exactly to *M*, only to M−α(M), where α(M) is some function with

$$\lim_{M\to \infty }\alpha \left(M\right)/M=0.$$

The function α(M) is typically of the order of logM, and it gives us a little freedom to simplify expressions by ignoring logarithmic terms. For any cell Γ, let

$$\begin{array}{c} \mathrm{Boltz}(\Gamma )=\log L(\Gamma ) \end{array}$$

Let C(d,M) be the union of all cells Γ with

Boltz(Γ)<M−d.

We are looking for an entropy increase property saying the following. If we start from any cell Γ, then as time passes, the proportion of those of its elements that do not end up in a cell with entropy M−d becomes smaller than $\varepsilon_{d}$ where $\lim_{d\to \infty }\varepsilon_{d}=0$. In the formulation, we write $\varepsilon_{d}$ as $2^{-f_{1}\left(d\right)}$.

**Assumption** **1** (Boltzmann Entropy Increase Property)**.** *There is a constant $a_{1}>0$, a function $f_{1}\left(d\right)$ with $\lim_{d\to \infty }f_{1}\left(d\right)=\infty$ and a function $f_{2}\left(t,d,e\right)$ with $\lim_{t\to \infty }f_{2}\left(t,d,e\right)=f_{1}\left(d\right)$ such that for all d,e with $a_{1}\alpha \left(M\right)<d<e$ and Γ⊄C(e,M), we have*

$$L\left(U^{t}\Gamma \cap C\left(d,M\right)\right)/L\left(\Gamma \right)<2^{-f_{2}\left(t,d,e\right)}.$$

Below, we spell out two conditions that would imply the entropy increase formulated above. The first one says the union C(d,M) of all “small” cells taken together is small. We call it the Noncompensation Condition to suggest that the smallness of small cells is not compensated by their quantity. The condition holds for typical systems simply because the total number of cells is small.

**Assumption** **2** (Noncompensation Condition)**.** *There are constants $b_{1},b_{2}$ such that for $b_{1}\alpha \left(M\right)<d$, we have*

$$L\left(C\left(d,M\right)\right)/L\left(\Omega \right)\leq 2^{-b_{2}d}.$$

In Example A2, the total number of cells is the total number of partitions of *n* into $\sum_{i=1}^{m}n_{i}$, which is less than $n^{m}$; therefore, $L\left(C\left(d,M\right)\right)/L\left(\Omega \right)\leq 2^{-(d-m\log n)}$. Typically, we will have M>n, and we agreed that $m<n^{1/2}$. So, if we choose $\alpha \left(M\right)=M^{1/2}\log M$, then if d>2α(M), we have $d-m\log n>d-n^{1/2}\log n=d-\alpha \left(M\right)>d/2$, so the condition holds with $b_{1}=2$, $b_{2}=1/2$.

**Example** **2.** *The enormous differences in cell sizes seem to be typical for statistical mechanical systems even without reference to the small number of cells. Let, for example, in a container of a mole of gas, our macroscopic variable be the binary number $0.\omega_{1}\omega_{2}\ldots \omega_{n}$, giving approximately the relative quantity of gas in the left half of the container. Then, the cells $\Gamma_{0.000}$ and $\Gamma_{0.011}$ are absolutely negligible in volume compared to the cells $\Gamma_{0.001}$ and $\Gamma_{0.010}$.*

Algorithmic coarse-grained entropy satisfies the Noncompensation Condition automatically with $b_{2}=1$. Indeed, the set $\left\{\;\omega :H^{n}\left(\omega \right)<m\;\right\}$ is contained in $E_{m}=\left\{\;\omega :H\left(\omega \right)<m\;\right\}$, which, according to (5), has a volume at most of $2^{-m}L\left(\Omega \right)$.

The second condition says that if the system starts from a state in a not very small cell, then after a time *t*, it is unlikely to end up in any small union of cells.

**Assumption** **3** (Weak Mixing Condition)**.** *There is a constant $c_{1}>0$, a function $h_{1}\left(d\right)$ with $\lim_{d\to \infty }h_{1}\left(d\right)=\infty$ and a function $h_{2}\left(t,d,e\right)$ with $\lim_{t\to \infty }h_{2}\left(t,d,e\right)=h_{1}\left(d\right)$ such that for all d,e with $c_{1}\alpha \left(M\right)<d<e$, if Γ⊄C(e,M) and D is a union of cells with $L\left(D\right)/L\left(\Omega \right)\leq 2^{-d}$, then*

$$L\left(U^{t}\Gamma \cap D\right)/L\left(\Gamma \right)<2^{-h_{2}\left(t,d,e\right)}.$$

This property says that as *t* grows, the transformation $U^{t}$, while preserving the volume of the cell Γ, will distribute its content thinly over a large area so that only a small fraction of it can intersect the small union of cells *D*. In other words, after a while, the cells will be “mixed”. The property is in general difficult to prove but is plausible in typical physical systems. The following theorem only serves to check out the consistency of the above concepts.

**Theorem** **8.** *The Weak Mixing Condition and the Noncompensation Condition imply the Boltzmann Entropy Increase Property.*

**Proof.**  Assume that the Weak Mixing Condition and the Noncompensation Condition with the appropriate constants and functions $a_{1},b_{1},b_{2}$, $h_{1},h_{2}$, and let Γ⊄C(e,M). Let D=C(d,M) and $d>b_{1}\alpha \left(M\right)$; then, the Noncompensation Condition implies $L\left(D\right)/L\left(\Omega \right)\leq 2^{-b_{2}d}$. The Weak Mixing Condition implies

$$L\left(U^{t}\Gamma \cap D\right)/L\left(\Gamma \right)<2^{-h_{2}\left(t,b_{2}d,e\right)}$$

for *d* such that $c_{1}\alpha \left(M\right)<b_{1}d$. Thus, the Boltzmann Entropy Increase Condition holds with $a_{1}=c_{1}/b_{1}$ and $f_{2}\left(t,d,e\right)=h_{2}\left(t,b_{2}d,e\right)$. □

The following examples illustrate the insufficiency of the traditional Boltzmann entropy logL(Γ).

**Example** **3.** *Take a large container filled with ideal gas and a few large balloons. At the start, the balloons are fixed. Then, we release them. They gain energy from collisions with the gas molecules until they achieve the average energy appropriate to their number of degrees of freedom. It is reasonable to count the positions of the balloons to the macrostate of the system. The volume of the cell will be essentially determined by the energy of the system consisting of the gas alone. This energy, and hence the Boltzmann entropy, becomes smaller by the amount transferred to the balloons.*

This example becomes less ridiculous if we replace balloons by tiny colloid particles, like in the Brownian motion, or by the memory of a computer. For a while, it will still be reasonable to count the content of the memory as part of the macroscopic description: it is given by specifying the “global” charges and magnetizations of all the tiny areas on the disks and the silicon memories. However, as the size of a site storing an individual bit decreases, there will come a point where it is not reasonable to consider the memory state as part of the macroscopical description. The communication of two computers, one with “macroscopic” memory and the one with “microscopic” memory, leads to the Maxwell demon paradox. This shows the necessity of the smooth transition between macroscopic and microscopic transitions exhibited by algorithmic entropy.

In terms of our scheme, we are talking about increasing *n* (refining the partition). The additive term $H(\omega^{n})$, which is so insignificant for small values of *n*, gains in significance in this process and makes the transition continuous. Ignoring it, and defining entropy just as $\log L(\omega^{n})$, is bound to lead to paradoxes.

The following system also defies Boltzmann entropy but submits to algorithmic coarse-grain entropy.

**Example** **4** (The baker’s map)**.** *Let* Ω *be the set of doubly infinite binary sequences $\omega =\ldots \omega_{-1}\omega_{0}\omega_{1}\omega_{2}\ldots$ with the shift transformation ${\left(U^{t}\omega \right)}_{i}=\omega_{i+t}$ over discrete time. Let us write $\omega^{n}=\omega_{-\left\lfloor n/2\right\rfloor }\cdots \omega_{\left\lceil n/2\right\rceil -1}$. The n-cells have the form $\Gamma_{\omega^{n}}$. Let the volume L be such that all n-cells have the same volume $2^{-n}$.*

Since all *n*-cells have the same measure no matter what fixed precision we choose, the Boltzmann entropy of $U^{t}z$ does not increase with *t*. The quantity $H^{n}\left(U^{t}z\right)$, however, will be shown below to increase fast for all typical sequences, between times time 0 and *n*, linearly from −n to 0.

Consider a typical example of an infinite sequence *z* which has $z^{n}=0\cdots 0$ and whose other bits are random (this has now a precise meaning, but let us just use the informal understanding). Then, $K(z^{n})\leq 2\log n$, $\log\Gamma_{z^{n}}=-n$; therefore,

$$H^{n}\left(z\right)\leq 2\log n-n.$$

Now, for t>n, the string ${\left(U^{t}z\right)}^{n}$ consists of random bits, so essentially, $H^{n}\left(U^{t}z\right)\geq 0$. Between time 0 and *n*, this algorithmic coarse-grained entropy increases linearly from −n to 0. The fine-grained algorithmic entropy $H(U^{t}z)$ will, on the other hand, only increase slowly, since our choice of the precision n+t can follow the increase of *t*:

$$\begin{array}{cc} H(U^{t}z) & \overset{+}{<}H^{t+n}\left(U^{t}z\right)\leq 2\log n+2\log t+t-\left(t+n\right)\\ & =2\log n+2\log t-n. \end{array}$$

On the right-hand side of the first equation, the first two terms upper-bound the complexity of *n* and *t*, while the second term is the length of the nonzero part of the string ${\left(U^{t}z\right)}^{t+n}$, so the first three terms bound $K({\left(U^{t}z\right)}^{n+t})$. The last term gives the logvolume of the cell. This inequality shows that $H(U^{t}z)$ can only grow as slowly as logt. The general inequality (17) gives $H\left(U^{t}\omega \right)-H\left(\omega \right)\overset{+}{<}I\left(t:\left(U^{t}\omega \right)\right)$. For rational values of *t*, the inequality (11) gives $I\left(t:\left(U^{t}\omega \right)\right)\overset{+}{<}H\left(t\right)$. Hence,

$$H\left(U^{t}\omega \right)-H\left(\omega \right)\overset{+}{<}H\left(t\right).$$

This means that for some simple rational values of *t*, the algorithmic entropy hardly increases at all. For example, if *t* is an integer of the form $2^{n}$, then the increase is at most 2loglogt.

We can also use independent biased coin tossings for the measure, where the probability of 1 is p=0.3. If $\omega^{n}=0\cdots 0$, then $\mathrm{Boltz}(\omega^{n})=n\log0.7$. But for a random $\omega^{n}$, this value will be ≈n(0.3log0.3+0.7log0.7), which is considerably smaller. Therefore, the Boltzmann entropy decreases strongly. The algorithmic Boltzmann entropy is, on the other hand, negative for the all 0 starting cells and grows to ≈0 as expected.

We can even use any ergodic stationary process for the measure and obtain similar results. For such processes, there is an “asymptotic equidistribution property” guaranteeing that the most volume will be taken up by *n*-cells of about the same size $2^{-hn}$ (where *h* is the so-called “entropy rate”).

In typical physical systems, the partitions given by the canonical cells have no simple connection with the computable transformation group $U^{t}$ of our dynamical system. In particular, they are not “generated” from the first partition into $\Gamma_{0}$ and $\Gamma_{1}$ by $U^{t}$ the way they are in the baker’s map.

**Remark** **2** (Kolmogorov–Sinai entropy)**.** *An arbitrary ergodic computable stationary measure could be considered in place of the coin tossing as well. Let us point out the connection of our quantities to the Kolmogorov–Sinai entropy of such a measure. For a random sequence (and hence for almost all sequences) ω, the algorithmic Boltzmann entropy $K\left(\omega^{n}\right)+\log\mu \left(\Gamma_{\omega^{n}}\right)$ remains bounded. This quantity is the difference of two quantities that increase therefore equally fast: $K(\omega^{n})$ and $-\log\mu (\Gamma_{\omega^{n}})$. According to the Shannon–McMillan–Breiman Theorem, with probability 1, we have $\lim_{n\to \infty }-\log\mu \left(\Gamma_{\omega^{n}}\right)/n=h$ where h is the Kolmogorov–Sinai entropy of the process μ. Thus, the Kolmogorov–Sinai entropy shows the rate of increase in the Boltzmann entropy of a stationary process as the partition is being refined. Due to the boundedness of the quantity $K\left(\omega^{n}\right)+\log\mu \left(\Gamma_{\omega^{n}}\right)$, we arrive to (a version of) Levin’s theorem from [14], saying that this is also the rate of increase of $K(\omega^{n})$.*

The reason that Boltzmann entropy does not increase to logL(Ω) in the baker’s map with any stationary measure is because in case of the uniform distribution, the *n*-cells have the same volume, and in the more general case still, there is an “asymptotic equidistribution property” guaranteeing that most of the volume will be taken up by *n*-cells of about the same size $2^{-hn}$ (where *h* is the so-called “entropy rate”). Therefore, the Noncompensation Condition (the more trivial of the two conditions) is not satisfied. If for this same map we use *n*-cells of different enough volumes to satisfy this condition, then the Boltzmann entropy will increase. In fact, when $H^{n}\left(\omega \right)$ is high, this can be treated as a concise expression of the fact that in every “simple” partition with extremely different cell sizes, the point ω would end up in a large cell.

The example of the shift transformation suggests that in typical chaotic systems, the parameter *n* can be made a function of *t* as long as it grows slower than linearly with *t*. Thus, if $\lim_{t\to \infty }n\left(t\right)/t=0$, then in the baker’s map with the uniform distribution,

$$H^{n(t)}\left(U^{t}\omega \right)$$

will approach logμ(Ω) almost as fast as if we held *n* constant. The growth of n(t) seems a good measure of the mixing of $U^{t}$.

In conclusion, we suggest that the new quantity $H_{L}^{n}\left(\omega \right)$ extends the idea of entropy increase to a wider class of chaotic systems than the one in which it has originally worked, and it can also serve as a useful tool for formulating conjectures concerning the nature of chaoticity and its extent.

### 6.4. The Paradox of Typicality

The notion of a typical object is an informal one, and here we call attention to the fact that our intuition concerning the properties of typical objects may be misleading. Consider the space of infinite 0–1 sequences obtained by tossing a biased coin with probabilities 1/3, 2/3. We would consider typical those sequences in which the relative frequency of 0s tends to 1/3. On more reflection, we would consider those sequences ω typical that also satisfy all other criteria or randomness and are random according to Martin–Löf’s definition, or, equivalently, which have $H_{p}\left(\omega \right)>-\infty$ where *p* is the appropriate coin-tossing measure.

Consider now a dynamical system with the volume measure *L*. The (fine-grained) entropy nondecrease property, which can also be considered a randomness-conservation property, guarantees that the above-defined typicality is “conserved”: the evolution of a system takes typical states into typical ones.

There is, however, another similarly attractive idea of typicality, which we will call “local typicality”, which is not conserved. Consider a given partition $P_{n}$ and a given cell Γ in this partition. Let us call those states ω of the cell Γ “locally typical” whose fine-grained entropy $H_{L}\left(\omega \right)$ is close to the coarse-grained entropy $S(\omega^{n})$. We know from the Stability Theorem 5 that most points of each cell (in terms of the measure *L*) are typical in this sense. However, local typicality is not conserved. Indeed, assume that coarse-grained entropy increases for most points and that the coarse-grained entropy of Γ is low. Then, for most points ω in Γ (and hence for most locally typical points), their coarse-grained entropy increases, so $U^{t}\omega$ belongs to a cell $\Gamma^{\prime }$ with much higher coarse-grained entropy. At the same time, the fine-grained entropy $H_{L}\left(U^{t}\omega \right)$ does not change too much with respect to $H_{L}\left(\omega \right)$. So, the locally typical state ω turns into a locally nontypical state $U^{t}\omega$. The reason is that ω was a locally typical point of a “nontypical” cell, and it is still carrying this history. However, as long as only the macroscopic information embodied in the cell $\Gamma^{\prime }$ is available for inspection and manipulation, this history is inaccessible to later observers.

The non-conservation of local typicality eliminates a potentially attractive “principle”: namely that the state we have at present is locally typical. Consider the present state of a container of gas after a wall was removed that had confined the gas to one half. In a usual macroscopic description (partition), the coarse-grained entropy of the present state will be much larger than what it was before the wall removal. Since the fine-grained entropy is approximately the same (since it did not change much), the present state is actually highly nontypical locally.

The refuted principle is attractive since, together with the Boltzmann Entropy Increase Property (or its counterpart using algorithmic coarse-grained entropy), it could be used to prove that an entropy increase is not only likely to occur but will occur. One possible substitute of the principle is the introduction of probabilistic perturbations, see e.g., [15]. We prefer to say the following:

The entropy increase property relates strictly only to the present macroscopic state of our system, and it does not assert directly anything about the present microscopic state.

## 7. Maxwell’s Demon

### 7.1. Entropy Balance

Let X and Y be two systems where Y is considered to be the environment from which X is temporarily isolated. In order to to “do something” to X, we couple it with Y, giving rise to a joint Hamiltonian and a joint transformation $U^{t}\left(\xi ,\eta \right)$. Let us assume that, being in classical mechanics, the impulses and momenta of the joint system are simply the impulses and momenta of the two subsystems; therefore, the Liouville measure on X×Y is, even in the coupled system, the product of the original Liouville measures $L_{X},L_{Y}$ in the subsystems. Let $\left(\xi_{t},\eta_{t}\right)=U^{t}\left(\xi ,\eta \right)$, and

$$\Delta H\left(\xi \right)=H\left(\xi_{t}\right)-H\left(\xi \right),$$

where, for clarity, we omitted the subscripts $L_{X},L_{Y}$. Notice now that for our measure,

H(ξ)+H(η)=H(ξ,η)+I(ξ,η),

using the definition (8) for I(ξ,η).

**Theorem** **9** (Entropy Balance)**.**

$$\Delta H\left(\xi \right)+\Delta H\left(\eta \right)\overset{+}{>}I\left(\xi_{t},\eta_{t}\right)-I\left(\xi ,\eta \right)-I\left(t:\xi ,\eta \right).$$

**Proof.**  According to (17) applied to the joint system, we have $\Delta H\left(\xi ,\eta \right)\overset{+}{>}-I\left(t:\xi ,\eta \right)$. (This formula cannot be applied now to the parts of the system since they do not have their own transformations now.) Using this, we have

$$\begin{array}{cc} H\left(\xi_{t}\right)+H\left(\eta_{t}\right) & =H\left(\xi_{t},\eta_{t}\right)+I\left(\xi_{t},\eta_{t}\right)\\ & \overset{+}{>}H\left(\xi ,\eta \right)-I\left(t:\xi ,\eta \right)+I\left(\xi_{t},\eta_{t}\right)\\ & =H\left(\xi \right)+H\left(\eta \right)+I\left(\xi_{t},\eta_{t}\right)-I\left(\xi ,\eta \right)-I\left(t:\xi ,\eta \right), \end{array}$$

which gives the statement by rearrangement. □

Since the last term is generally negligible, this theorem says that if the two systems were originally independent (I(ξ,η)≈0), then a decrease in the entropy of ξ must be accompanied by an increase in the entropy of η. The entropy balance theorem is not new, of course, for Boltzmann entropy. But its present form makes it useful for the treatment of Maxwell’s demon.

### 7.2. Maxwell’s Demon and Landauer’s Thesis

Maxwell’s demon is a being sitting at a tiny door between two compartments of gas and letting the molecules through selectively with the goal of entropy decrease in the container. Principles of thermodynamics seem to contradict the possibility of such a demon, so it seems paradoxical and demanding explanation. The typical explanations assume either that the door will heat up and begin to work randomly after a while or that in order to make its observations, the demon must descend into this world more than she cares to and interact energetically with the molecules; this heats her up, making it harder and harder for her to concentrate. These explanations introduce additional physical assumptions which are alien to the general mathematical nature of the second law (increase of disorder). Several such explanations are refuted by more refined models (see [16]).

A convincing modern solution emerged in a principle announced by Landauer (see [16]). Let us model the demon as some computer-controlled device interacting with the gas. She seems to be able to decrease the Boltzmann entropy of the gas only at the expense of the increase in her own information content. Landauer introduced a principle saying that in order to erase a bit of information, a certain minimal amount (kTlog2) of heat dissipation into the environment (and, of course, investment of the corresponding amount of work into the system) is necessary.

**Remark** **3.** *In order to prove that the erasure results in heat dissipation, Landauer argues that the erasure must be a general operation that decreases the phase space of the computer memory. I find it difficult to interpret the increase in H(η) universally as heat dissipation. Consider a memory ξ consisting of a row of pendulums swinging transversally. The bit 1 means that the pendulum swings, while the bit 0 means it does not. Let the “environment” η be an identical row of pendulums, each of which hangs motionless originally. Now, we can use η to erase the memory by moving it next to ξ in the right moment. The change in η is obviously reversible, so there is no heat dissipation.*

Using our framework, the demon paradox occurs since the state of the demon’s memory was implicitly considered part of the macroscopic description of the joint gas–demon system: the quantity of information in it failed to contribute to the classical Boltzmann entropy. Since this device is able to decrease the Boltzmann entropy of the gas at the expense of the increase in the information content of its memory (without increasing its own Boltzmann entropy), it is able to decrease the Boltzmann entropy of the total system. Our solution eliminates the paradox by including the information content (complexity) of the macroscopic description into the expression for entropy. It should be considered as the rigorous formulation of the more special and informal principles of Landauer and Zurek.

Zurek [7] saw that Maxwell’s demon paradox and Landauer’s thesis are two sides of the same interaction between an information-processing machine (the demon) and a classical thermodynamic system. The demon turns entropy into information, while the information-erasure operation turns information into entropy. Zurek constructed an entropy-like quantity specifically for this situation and argued that it is nonincreasing. He created a special macroscopic variable *d* (without actually distinguishing macroscopic and microscopic) whose value is equal to the demon’s memory state. He defined then a quantity called “physical entropy” associated with such a system that is essentially Z(a,d)=Boltz(a)+K(d) where Boltz(a) is the Boltzmann entropy of the classical part. This can be seen as essentially the same as Boltz(a,d)+K(a,d). Indeed, Z(a,d)=Boltz(a), since the demon’s macroscopic and microscopic states are the same. Also, we can delete *a* from K(a,d), since we are interested in situations in which *d* contains much more information than *a*.

Zurek argues that if at constant temperature *T*, the system is brought from state $\left(a_{1},d_{1}\right)$ to state $\left(a_{1},d_{2}\right)$, then the amount of work obtained is at most $Z\left(a_{2},d_{2}\right)-Z\left(a_{1},d_{1}\right)$. For this, he tacitly assumes that the work gained from the operation of the system can be separated into the work obtained from bringing the classical machine from $a_{1}$ to $a_{2}$ and the work bringing the memory from $d_{1}$ to $d_{2}$. With this assumption, the second law indeed implies the upper bound $T(\mathrm{Boltz}\left(a_{2}\right)-\mathrm{Boltz}\left(a_{1}\right))$ on the first kind of work, and Landauer’s principle implies the bound $T(H\left(d_{2}\right)-H\left(d_{1}\right))$ for the second kind of work.

Formally, our coarse-grained algorithmic entropy looks similar to Zurek’s but is defined more generally, and it has many connections to various different definitions of entropy (for ensembles as well as cells) and also to the theory of randomness. In particular, the above bound can be proven without the tacit assumptions.

Let ξ be the gas whose entropy the demon is trying to decrease. We also count the whole state η of the demon into her macroscopic description. As it is usual with classical machines, we can assume that there is an *n* such that $H^{n}\left(\xi \right)$ is close to H(ξ) but *n* (and therefore $K(\xi^{n})$) is still negligibly small with respect to H(ξ) and therefore

$$H\left(\xi \right)\approx \log L\left(\Gamma_{\xi^{n}}\right).$$

The Entropy Balance Theorem guarantees that going from (ξ,η) to $\left(\xi^{t},\eta^{t}\right)$, the decrease in the sum H(ξ)+H(η) will be small. Since $H^{n}\left(\xi^{t}\right)\overset{+}{>}H\left(\xi^{t}\right)$, this implies that any decrease in $H^{n}\left(\xi \right)$ (which is now essentially the Boltzmann entropy of the machine) must be compensated by an increase in H(η)≈K(η), which is the information content of the demon’s memory (this is the Maxwell’s demon direction) and vice versa (this is the Landauer thesis direction).

## Data Availability

No new data were created or analyzed in this study.

## Appendix A. Ten Minutes on Classical Thermodynamics

### Appendix A.1. Thermodynamical Systems

Entropy was first introduced in classical, “phenomenological” thermodynamics. This theory, which is also the form of thermodynamics most widely used in engineering, is concerned with a physical system when the latter is in a state called “equilibrium”.

An *equilibrium state* of the system is characterized by the fact that for all practical purposes, its properties relevant for interaction with the rest of the world are determined by a relatively small number of parameters (functions of the state) called *macroscopic* parameters $u_{1},\ldots ,u_{m}$. The simplest system to consider is a certain quantity of gas in a container with just a few macroscopic parameters: volume, temperature energy and pressure. Two of these can actually be deleted, since they are a function of the other two, but it is not necessary for our purposes to minimize the number of parameters.

Let us agree that energy will always be included among the parameters. The *first law of thermodynamics* is a consequence of a more general law of physics: it says that the energy of an isolated system does not change. The interaction of the system with the outside world involves some “exchange of energy”: in other words, the energy of the system plus its environment cannot be expressed by a simple sum of the two energies.

### Appendix A.2. Dynamics and Volume

According to the laws of classical mechanics, an isolated system undergoes an evolution described by a transformation group $U^{t}$. If at time $t_{0}$, the system was in state ω, then at time $t+t_{0}$, it will be in state $U^{t}\omega$. The group $U^{t}$ is generally given by a system of differential equations which, at least in the example of ideal gas given with coordinates and impulses, are called the Hamiltonian equations. For this case, Liouville’s Theorem holds, saying that the volume of a domain remains invariant under transformation under $U^{t}$. In most other cases also, a natural measure is found on Ω that remains invariant under $U^{t}$; we will call this measure the *volume* and denote the volume of a set *A* by L(A).

The law of energy conservation says that during the evolution of an isolated system, it is confined to a surface in the state space determined by the requirement that the energy is equal to a certain value. Therefore, the volume measure to use will be actually obtained by restricting the original volume measure to a thin layer determined by the requirement that the value of energy is in a certain small interval and normalizing. This measure, in the limit, is called the *microcanonical ensemble*.

### Appendix A.3. The Paradox of Irreversibility

If a mechanical system has a certain trajectory $t\mapsto U^{t}\omega$ of its evolution from state ω=a to state $U^{t_{1}}\omega =b$, then there is also a trajectory of evolution from state *b* to state *a*. It is sufficient to reverse all the final velocities of all particles, and the system will trace its trajectory backward. Therefore, any evolution seems just as possible as the corresponding reverse evolution.

At the same time, the world seems to be full of irreversible phenomena. Imagine a container *A* of gas separated from an empty container *B* by a wall. After the removal of the wall, much of the gas will occupy container *B*, and the reverse physical process, when the gas collects itself spontaneously in part *A*, will never be seen. The reversibility of the equations seems to be in contradiction to irreversibilities of this kind.

In the main part of the paper, we explain how coarse-graining resolves the paradox.

### Appendix A.4. Equilibrium

For an isolated system, we may want to call *equilibrium states* those macrostates that are relatively stable: the values of the macroscopic variables will undergo only small fluctuations in time. On reflection, however, this requirement must be weakened to hold only for *most* microstates within the macrostate (as measured by volume).

How can an equilibrium state be transformed into a different equilibrium state at all? Suppose that our system is a container of gas. The system can be combined with some other systems like a heat reservoir or a piston connected to a lever. Then, some constraint is removed (e.g., an insulation is removed or a piston is allowed to move), changing the nature (the equations of motion) of the joint system by making its parts interdependent. When a new equilibrium is reached, the constraints can be restored.

A useful formal way to describe a nonisolated equilibrium system is via an *ensemble* (probability distribution) invariant in time. Such a system does not have a deterministic evolution describable by a transformation $U^{t}$, since the evolution of the system ω is only the projection of the (possibly deterministic) evolution of a larger system (ω,ξ). The most popular ensemble for such cases is the Gibbs *canonical ensemble* (a generalization of the so-called Boltzmann distribution) defined by a density proportional to $e^{-E(\omega )/kT}$ with the energy E(ω), the Boltzmann constant *k* and the temperature *T*. It can be shown to be invariant if the system ω is part of a large system (ω,ξ) where ξ is a heat reservoir of temperature *T*.

### Appendix A.5. Boltzmann Entropy

Boltzmann defined the *entropy* of a macroscopic state *a* as the logarithm of the volume of $\Gamma_{a}$:

(A1) $$\mathrm{Boltz}\left(a\right)=\log L\left(\Gamma_{a}\right).$$

The most obvious problem with this definition seems to be its dependence on the particular choice and number of macroscopic variables and the precision with which we want to determine them. Indeed, another digit of precision will decrease the Boltzmann entropy of most states by about log10. The volumes in question are, however, in the typical classical examples, so large, that such a small difference is negligible (especially if we take into consideration that the actual definition also multiplies Boltz(a) by the very small Boltzmann constant *k*). We will also use the notation

$$\mathrm{Boltz}\left(\Gamma_{a}\right)=\mathrm{Boltz}\left(a\right)$$

since it is harmless here to identify a cell with its description.

**Example** **A1.**
*Let us consider n identical, indistinguishable molecules of gas in a container with rigid walls and the following cell of phase space. The positions of the molecules are restricted to the container of volume V. The total kinetic energy of the molecules is confined between the values K and K−ΔK. The kinetic energy is $\frac{1}{2m}\sum_{i=1}^{3n}p_{i}^{2}$ where $p_{i}$ represents the impulses $mv_{i}$ and m represents the mass of a molecule.*

The entropy for this “cell” consists of two terms. The first term is coming from the amount of freedom in choosing the positions of the molecules and is nlogV. The second term is the log volume of the set of points $p=(p_{1},\ldots ,p_{3n})$ such that $K-\Delta K\leq \frac{1}{2m}\sum_{i}p_{i}^{2}<K$. For “reasonable” values of ΔK, this is about the same as the log volume of the set of impulse points *p* with total energy <K, because most of the volume of a high-dimensional ball of *p* with $\sum_{i}p_{i}^{2}<2mK$ is near its surface. This volume is $C_{3n}{\left(2mK\right)}^{3n/2}$, where $C_{n}$ is the volume of an *n*-dimensional unit ball. This gives for entropy the value

(A2) $$\begin{array}{c} n\left(\log V+\frac{3}{2}\log\left(2mK\right)\right)+\log C_{3n}. \end{array}$$

It turns out that the correct volume measure to use is n! times smaller due to the fact that the molecules are not distinguishable. Therefore, logn! must be subtracted from here to obtain the correct value. This can be best seen when we consider the act of mixing two quantities of gas with the same pressure and temperature. Depending on whether the two gases are different kinds or not, the entropy of the mixture will or will not be greater than the sum of the two original entropies. (This is the so-called Gibbs paradox).

**Example** **A2.**
*Consider a container of ideal gas consisting of n molecules of the same kind which is partitioned, in our mind, into m compartments $C_{1},\ldots ,C_{m}$ where m is much smaller than n: say,*

$$m<\sqrt{n}.$$

*Ignore velocities for simplicity. Let the macro variable $n_{i}$ give the number of molecules in compartment $C_{i}$. Let $n=(n_{1},\ldots ,n_{m})$. Let us also consider a second set of variables, the numbers $i_{1},\ldots ,i_{n}$ telling that molecule j is in compartment $i_{j}$. The description $i=(i_{1},\ldots ,i_{n})$ is, of course, more detailed than the description n, which is a function n(i) of i. Therefore, $\Gamma_{n}={⋃}_{n=n(i)}\Gamma_{i}$.*

Since the molecules are all of the same kind, the dynamics, and therefore the measure *L* will certainly be invariant with respect to the exchange of molecules. Therefore, if $n\left(i\right)=n\left(i^{\prime }\right)$, then $L\left(\Gamma_{i}\right)=L\left(\Gamma_{i}^{\prime }\right)$. Let us consider first the molecules distinguishable. Then, $L\left(\Gamma_{n}\right)=N\left(n\right)p_{n}$ where $p_{n}$ is the common volume of all $\Gamma_{i}$ with n(i)=n, and

$$N\left(n\right)=\frac{n!}{n_{1}!\ldots n_{m}!}.$$

If we also assume that all the compartments have the same shape and size, then we obtain that $p_{n}$ does not depend on n. Indeed, $p_{n}=V_{m}^{n}$, which is the *n*-th power of the volume of a single compartment, since after i is given, what is left to determine is only the exact position of each molecule in its compartment. The Boltzmann entropy of state n is equal to

$$H\left(n\right)=\log L\left(\Gamma_{n}\right)\approx n\left(-\sum_{j}f_{j}\log f_{j}+\log V_{m}\right)$$

where $f_{j}=n_{j}/n$. Again, if the molecules are indistinguishable, it is necessary to subtract logn!.

Let us call a system *volume-defined* (this is the kind of system given preference in textbooks) if the two macroscopic variables *V* (volume) and *E* (energy) determine the rest. Here, *V* can be given exactly, and *E* is given with some precision. The entropy

S(V,E)

is given as the phase volume of the set of phase points corresponding to the coordinates in volume *V* and the energy between E−ΔE and *E*. Example A1 shows that this is approximately the volume of the set with the simple limitation that the energy is <E (the volumes of an *n*-dimensional ball and a shell of it are close).

Consider a system consisting of two parts 1,2, such that both parts have separate descriptions, which are both microscopic and macroscopic. Then, a cell corresponding to the joint system is the Cartesian product of its projection cells in systems 1 and 2, and its volume is also the product of the corresponding volumes. This way, the entropy of the joint system is the sum of the two entropies, so entropy is an *additive* function of the state.

Entropy was first defined in a so-called “phenomenological” model, using only the notions of temperature, heat and reversibility without reference to atoms. We will point out the connection more closely a little later, but the last example already gives some indication.

### Appendix A.6. The Growth of Entropy

Since entropy measures the amount of phase space occupied by a macrostate, it sounds plausible that a system tends to be in states with high entropy, but this is not obvious without further discussion. Anyway, this is exactly how classical thermodynamical systems are known to behave: according to one formulation of the *second law* of thermodynamics, entropy in isolated systems cannot decrease. There is also a “phenomenological” formulation, saying that heat cannot be transformed into work (leaving everything else unchanged). There are some other related entropy increase properties, so let us list all of those interesting us:

- In an isolated system, entropy cannot decrease;
- An isolated system undergoes an irreversible transformation exactly when its entropy actually increases;
- An equilibrium state of an isolated system is a maximum (or at least a local maximum) of entropy.

Just as the precise notion of reversibility had to be formulated statistically, the same can be expected for these entropy increase properties for Boltzmann entropy. Formulation and proof are, of course, different, and rigorous proof is unavailable for most realistic systems. This paper analyzes the heuristic arguments for the increase of Boltzmann entropy; here, we want to understand the ordinary physical consequences.

### Appendix A.7. Temperature and Pressure

Suppose that two volume-defined systems are brought into contact and are allowed to come into thermodynamic equilibrium without a change to their volume. Then, according to the maximum entropy principle, the joint entropy is maximal, so a small reversible energy exchange between the two containers should not change it. If at this time, therefore the amount of energy $dE=dE_{1}=-dE_{2}$ was transmitted reversibly from system 2 to system 1 and the entropy of system $S_{i}$ increased by $dS_{i}$ then $dS_{1}+dS_{2}=0$; it follows that

$$\frac{dS_{1}}{dE_{1}}=\frac{dS_{2}}{dE_{2}}.$$

We know from experience that two systems are in equilibrium only if their temperatures are the same, so we conclude that $\frac{dS}{dE}$ depends only on the temperature. In Example A1 of an ideal gas, all its energy is kinetic and therefore, using (A2), we have dS/dE=dS/dK=3n/2K. The quantity K/n is the average kinetic energy of a molecule, and $\frac{2}{3}\frac{K}{n}$ is sometimes used to define the *temperature T* of the (ideal, monatomic) gas. More generally, one can define temperature for volume-defined systems as follows. First, we express energy via entropy and volume as E(S,V). Then, we define

$$T=\frac{\partial E(V,S)}{\partial S}.$$

We can also introduce *pressure* for a volume-defined system by

$$p=\frac{-\partial E(V,S)}{\partial V}$$

obtaining the equation

dE=TdS−pdV

for reversible changes in volume-defined systems. Here, the first term is the heat part dQ of the energy received by the system, and the second one is the work part. The equation turns into inequality for irreversible changes.
